# Maternal smoking during pregnancy and the risk of congenital urogenital malformations: A systematic review and meta-analysis

**DOI:** 10.3389/fped.2022.973016

**Published:** 2022-10-03

**Authors:** Qiang Zhang, Zhi-Cheng Zhang, Xue-Yu He, Zhen-Min Liu, Guang-Hui Wei, Xing Liu

**Affiliations:** ^1^Chongqing Key Laboratory of Children Urogenital Development and Tissue Engineering, Chongqing Key Laboratory of Pediatrics, Ministry of Education Key Laboratory of Child Development and Disorders, National Clinical Research Center for Child Health and Disorders, China International Science and Technology Cooperation Base of Child Development and Critical Disorders, Children’s Hospital of Chongqing Medical University, Chongqing, China; ^2^Department of Urology, Children’s Hospital of Chongqing Medical University, Chongqing, China; ^3^Pediatric Research Institute, Children’s Hospital of Chongqing Medical University, Chongqing, China; ^4^Program for Youth Innovation in Future Medicine, Chongqing Medical University, Chongqing, China

**Keywords:** maternal smoking, congenital urogenital malformations, hypospadias, cryptorchidism, meta-analysis, paternal smoking

## Abstract

**Background:**

Investigations regarding the association between maternal smoking and specific urogenital teratogenesis exist. However, an integrated systematic review and meta-analysis studying the relationship by encompassing the whole urogenital system is essential.

**Objective:**

Even though many studies about inborn urogenital malformations have been conducted, its etiologic factors and exact pathogenesis are still unclear. Our aim is to assess the risk of congenital urogenital malformations in offspring of smoking pregnant women.

**Results:**

The meta-analysis, covering 41 case-control and 11 cohort studies, suggested that maternal smoking was associated with an increased risk of urogenital teratogenesis (odds ratio [OR] = 1.13, 95% confidence interval [CI]: 1.04–1.23, *p* = 0.005), cryptorchidism (OR = 1.18, 95%CI: 1.12-1.24, *p* = 0.0001), hypospadias (OR = 1.16, 95%CI: 1.01-1.33, *p* = 0.039), and kidney malformations (OR = 1.30, 95%CI: 1.14-1.48, *p* = 0.0001). Moreover, paternal smoking during the mother’s pregnancy was also significantly associated (OR = 1.26, 95%CI: 1.03-1.55, *p* = 0.028). The association between smoking > 10 cigarettes/day was evident but was not significant (OR = 1.24, 95%CI:0.81-1.88, *p* = 0.323).

**Conclusion:**

Our results showed that maternal smoking during pregnancy increased the risk of congenital urogenital malformations. In numerous epidemiological studies, maternal smoking during pregnancy has a significant role in fetal development. Therefore, quitting tobacco use may be an effective method for reducing the risk of congenital urogenital malformation in pregnant women.

## Introduction

The etiology of congenital malformations involves various genetic and environmental factors. However, the associations of environmental factors have been rarely characterized in the previous study (1). Maternal smoking, particularly during the gestation period, is a substantial risk factor for congenital malformations (2). Maternal smoking during pregnancy influenced the occurrence of congenital abnormalities (3). Due to the small prevalence of urogenital teratogenesis, a specific abnormality may be categorized as a genetic or gross defect (4). The mechanism of association between maternal smoking during pregnancy and congenital malformations has remained unclear. It was reported in several studies (5, 6) that maternal smoking during the gestation period was related to the increased risk of low birthweight (LBW) and spontaneous abortion. Regarding spontaneous abortion, the toxic effect of cigarette smoking during pregnancy on the fetus may cloud the teratogenic effect (7). Hence, the urogenital malformation rate of offspring among smoking pregnant women was relatively high (8). Moreover, the study (9) has shown that maternal smoking during pregnancy was significantly related to congenital urinary tract defects. Although the relationship between maternal smoking during pregnancy and specific urogenital malformations, such as urinary tract defects, cryptorchidism, hypospadias, and kidney defects in offspring seem to be established, an integrated systematic meta-analysis of the relationship considering the whole urogenital system is essential.

Maternal smoking during pregnancy is regarded as an adverse birth outcome. Tobacco smoke is composed of more than 2,000 compounds (8). Nicotine is a key ingredient in cigarettes. Also, nicotine replacement therapy has been proposed in countless studies. However, the safety and effectiveness of the method are unclear. The nicotine ingestion of pregnant women had a dose-response effect on the bloodstream of the embryo (10). As a result, the umbilical artery blood flow speed alters, affecting the fetal cardiovascular system (11). Nicotine, increases vasoconstriction and endothelial injury, causing hypoxia, which leads to abnormal fetal morphology (12). Hypoxia is associated with, elevated levels of environmental pollutants, and maternal smoking during pregnancy. Smoking during pregnancy is the most common fetal toxic exposure across the countries, which reduces fetal growth and increases the risk of some placental complications and fetal abnormalities (7, 13, 14).

Maternal smoking-associated teratogenesis may be related to hormone levels (15). Maternal smoking during pregnancy may cause metabolic derangement, that could contribute to urogenital teratogenesis (16). In male births, cryptorchidism and hypospadias are the most common urogenital malformations. Even though many studies about inborn urogenital malformations have been conducted, its etiologic factors and exact pathogenesis are still unclear. Many studies evaluated the interrelation between maternal smoking during pregnancy and specific urinary abnormalities. Still, there is no systematic review regarding the relationship between maternal smoking and urogenital teratogenesis. Therefore, we undertook this review and meta-analysis to evaluate the association of maternal smoking during pregnancy with the risk of congenital urogenital malformations.

## Methods

### Inclusion criteria

The studies that met the following criteria were selected: (1) the case group or the exposure group included babies diagnosed with congenital urogenital malformations; (2) the control group comprised babies without congenital urogenital malformations; (3) maternal smoking during pregnancy or paternal smoking during mother’s pregnancy or other words similar to smoking was investigated; and (4) the study was the cohort, case-control, or other designs.

### Exclusion criteria

The studies that met the subsequent criteria were excluded: (1) reviewed and repeated articles; (2) non-English and unable to access full articles; and (3) articles without providing valuable data.

### Search strategy

Guidelines for PRISMA (17) were followed in this study, and no similar research was found. In PubMed, Cochrane Library, Embase, Science Direct, and Web of Science Database, we searched for relevant, English-language studies until February 22, 2022. The search term was ((((((gestational smoking) OR gestational cigarette exposure) OR gestational tobacco exposure) OR nicotine exposure)) AND ((((((((congenital anomalies) OR Congenital Abnormality) OR Congenital Defects) OR Congenital Defect) OR Fetal Malformations) OR Fetal Malformation) OR Fetal Anomalies) OR Fetal Anomaly)). Moreover, we identified studies based on reference literature.

### Data extraction and quality assessment

**FIGURE 1:**
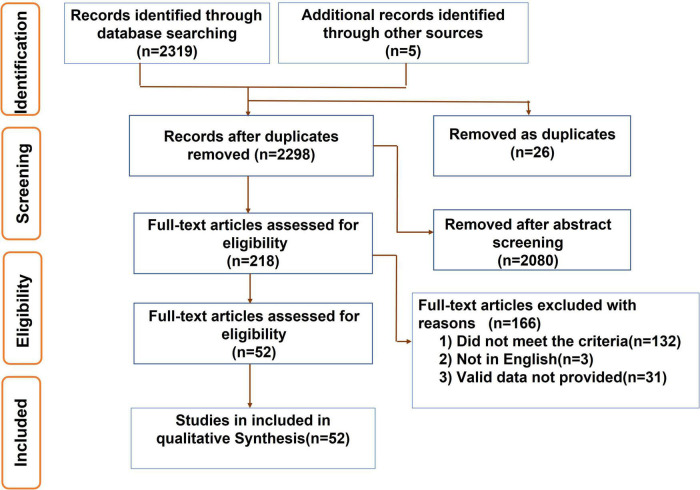
Flow diagram showing the process of study identification and included.

(Figure 1) displays flow diagram of the process of study identification and included. We assessed every selected study carefully. Two reviewers independently extracted the first author, year of publication, country, research type, number of congenital urogenital malformations, and maternal or paternal smoking during pregnancy, respectively in each group, as well as the count of cigarettes/day, type of congenital urogenital malformation, the ORs and CIs. The third author independently reviewed all of the selected articles. Additionally, we described the included study’s characteristics (Supplementary Table 1). Eventually, the quality assessment of studies was conducted based on the Newcastle-Ottawa Scale (NOS) (18).

### Statistical analysis

Due to diversity in the follow-up span, maternal or paternal characteristics, and inclusion criteria for case offspring, heterogeneity may occur in data analysis (19). Heterogeneity plays an essential role in statistical analysis and could lead to sampling errors (20). To assess heterogeneity between studies, the X^2^ test was used, as well as the I^2^ statistic of inconsistency. We combined data using the random effects model when there was heterogeneity, otherwise, we used the fixed effects model. Using existing data in included studies, we calculated the independent OR, ES, and 95%CI of the relationship between maternal or paternal smoking during pregnancy and the risk of congenital urogenital malformations. There was significance for *p*-values of < 0.05. An Egger’s test was conducted to assess publication bias, and the results were tested for stability using a sensitivity analysis. Calculations were made using STATA/MP 17.0 software (StataCorp, College Station, TX, United States).

## Results

**FIGURE 2:**
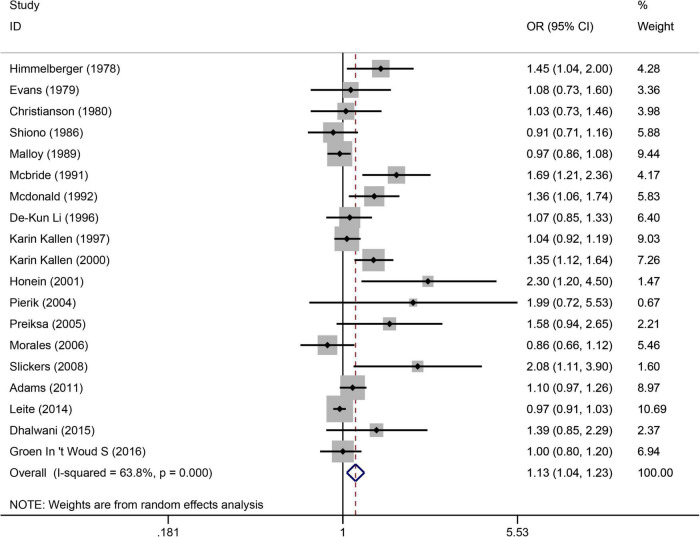
Forest plot for the relationship between maternal smoking during pregnancy and the risk of congenital urogenital malformations. Studies are sorted according to the sequence of publication time.

The following databases were searched: PubMed, Cochrane Library, Embase, Science Direct, and Web of Science Database, covering 41 case-control articles (1, 3, 4, 9, 15, 21–56) and 11 cohort articles (7, 57–66). (Figure 2), from the 19 articles (1, 3, 4, 7, 9, 24, 25, 30, 34, 38, 49, 52, 57–61, 64, 65), suggested that maternal smoking was associated with an increased risk of urogenital teratogenesis (OR = 1.13, 95%CI: 1.04–1.23, *p* = 0.005). We combined data using the random effects model when there was high heterogeneity (I^2^ = 63.8%). Based on the sensitivity analysis (Supplementary Figure 8), the results were stable. Using Egger’s test for publication bias, we found publication bias (*p* = 0.001). Perhaps, the results need careful interpretation.

### Maternal smoking during pregnancy and the risk of cryptorchidism

**FIGURE 3:**
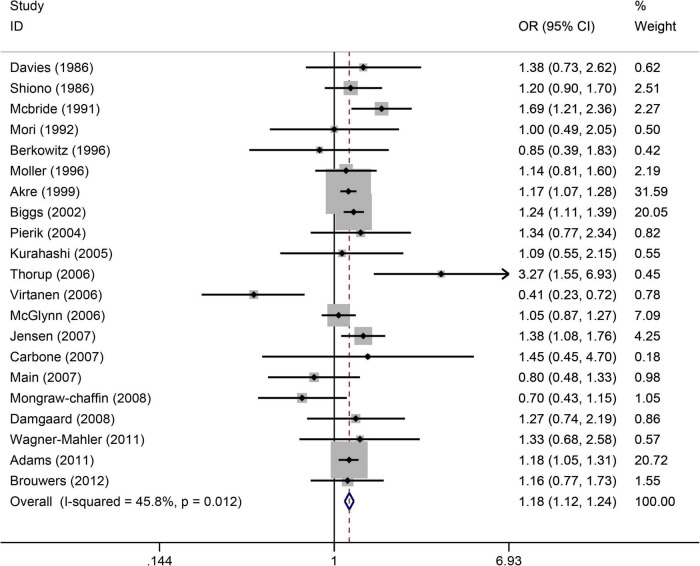
Forest plot for between maternal smoking during pregnancy and the risk of cryptorchidism. Studies are sorted by the sequence of publication time.

(Figure 3) shows the relationship between maternal smoking during pregnancy and the increased risk of cryptorchidism. A total of 21 studies (4, 22, 25–29, 31, 34, 36, 39, 40, 42–44, 46, 48, 52–54, 63) showed that maternal smoking during pregnancy increased the risk of cryptorchidism by 1.18 times (OR = 1.18, 95%CI:1.12–1.24, *p* = 0.0001). As there was no significant heterogeneity (I^2^ = 45.8%, *p* = 0.012), a fixed effects model was used. Based on Egger’s test (*p* = 0.689), there was free publication bias.

### Maternal smoking during pregnancy and the risk of hypospadias

**FIGURE 4:**
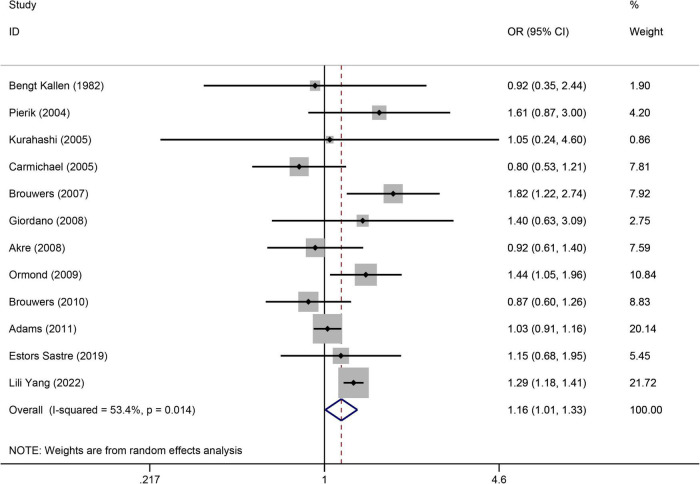
Forest plot for the relationship between maternal smoking during pregnancy and the risk of hypospadias. Studies were sorted by the sequence of publication time.

(Figure 4) displays the significantly increased risk of hypospadias associated with maternal smoking during pregnancy. There were 12 studies (21, 34, 35, 37, 41, 45, 47, 50–52, 55, 66) to evaluate the association between maternal smoking during pregnancy and the risk of hypospadias (OR = 1.16, 95%CI: 1.01–1.33, *p* = 0.039). The random effects model was used as there was significant heterogeneity (I^2^ = 53.4%). Sensitivity analysis (Supplementary Figure 9) showed the stability of the results. The Egger’s test was used to assess publication bias, which showed no evidence of publication bias (*p* = 0.712).

### Maternal smoking during pregnancy and the risk of kidney malformations

**FIGURE 5:**
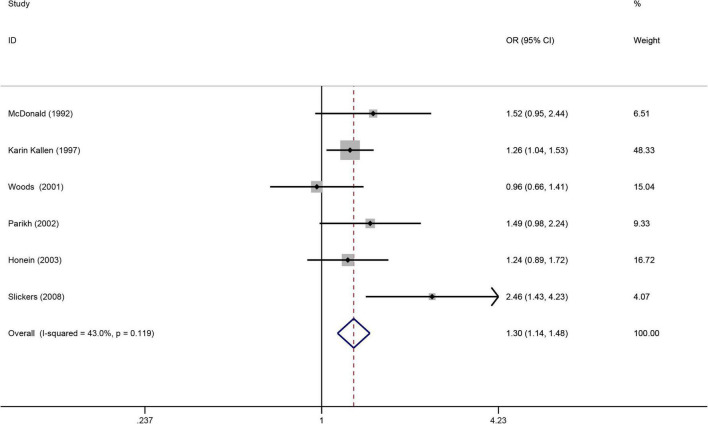
Forest plot for the relationship between maternal smoking during pregnancy and the risk of kidney malformations. Studies were sorted by the sequence of publication time.

(Figure 5) displays a significant increased risk of having a baby with kidney malformations in the six articles (32, 33, 49, 59, 60, 62) when maternal smoking during pregnancy (OR = 1.30, 95%CI: 1.14–1.48, *p* = 0.0001). Due to the heterogeneity (I^2^ = 43%), we used the fax effects model. We evaluated the publication bias using Egger’s test and found that there is no publication bias (*p* = 0.331).

### Paternal smoking during a mother’s pregnancy and the risk of congenital urogenital malformations

**FIGURE 6:**
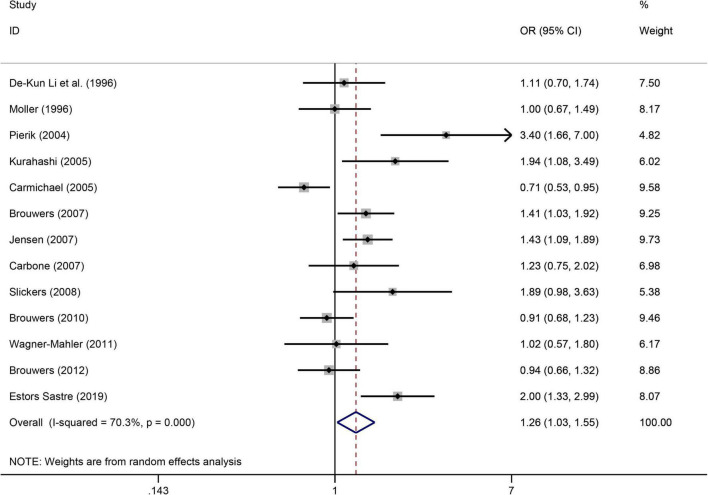
Forest plot for the increased risk of congenital urogenital malformations when paternal smoking during a mother’s pregnancy. Studies were sorted by the sequence of publication time.

(Figure 6) shows the significant association of risk of congenital urogenital malformations when paternal smoking during a mother’s pregnancy. A total of 13 case-control articles (9, 28, 34–36, 41–43, 49, 51, 53–55) showed that paternal smoking during a mother’s pregnancy increased the risk of congenital urogenital malformations by 1.26 times (OR = 1.26, 95%CI: 1.03–1.55, *p* = 0.028). Due to the high heterogeneity (I^2^ = 70.3%), we used the random effects model. A sensitivity analysis confirmed the robustness of the results (Supplementary Figure 10). We evaluated the publication bias using Egger’s test and found that there is no publication bias (*p* = 0.071).

### Amount of >10 cigarettes/day and the risk of congenital urogenital malformations

**FIGURE 7:**
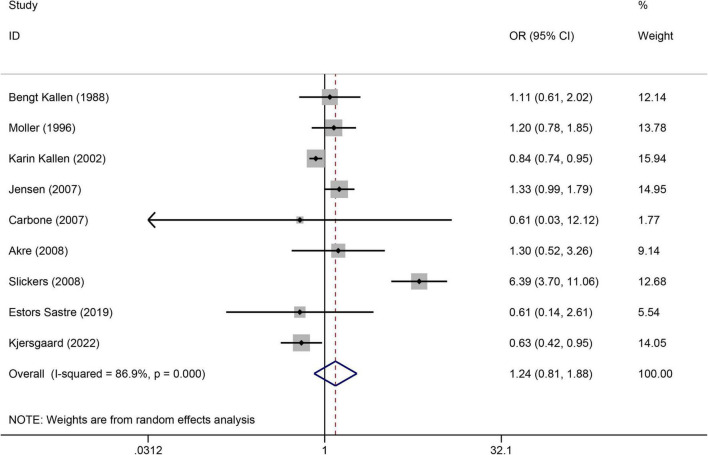
Forest plot for the non-significant association of > 10 cigarettes/day when maternal smoking during pregnancy. Studies were sorted by the sequence of publication time.

(Figure 7) presents the non-significant association of >10 cigarettes/day with maternal smoking during pregnancy. A total of 9 case-control articles (15, 23, 28, 42, 43, 45, 49, 55, 56) were used to evaluate the relationship between amount of >10 cigarettes/day and risk of congenital urogenital malformations (OR = 1.24, 95%CI:0.81–1.88, *p* = 0.323). We combined data using the random effects model when there was high heterogeneity (I^2^ = 86.9%). Using Egger’s test for publication bias, we found no publication bias (*p* = 0.331). A sensitivity analysis confirmed the stability of the results (Supplementary Figure 11).

## Discussion

With the growing incidence of urogenital teratogenesis, growing attention is directed toward the field of urogenital health. To our knowledge, the present study was the first systematic meta-analysis encompassing the whole urogenital system, that revealed an increased risk of urogenital teratogenesis in maternal smoking.

Our meta-analysis concluded that maternal smoking during pregnancy correlated with the increased risk of urogenital teratogenesis. The risk of genetic urogenital malformations in offspring of smoking pregnant women was found to be 1.13 times higher compared to non-smoking pregnant women. Maternal smoking over the gestation period was associated with an increased risk of cryptorchidism and hypospadias in the offspring. Moreover, we showed an increased association between paternal smoking during a mother’s pregnancy and urogenital teratogenesis. The included studies had statistical heterogeneity, and we conducted data analysis by using a random effects model. Moreover, sensitivity analysis showed that the results did not change before and after the analysis, which confirmed their stability.

The underlying mechanism of maternal smoking-induced congenital malformations remains unclear. Several studies have suggested possible mechanisms. The increased levels of carboxyhemoglobin in maternal and fetal blood are directly linked to fetal hypoxia and developmental defect associated with smoking during pregnancy (57). The teratogenic effect may be a sign of fetal hypoxia (67). In addition, the instability of natural killer cell activity and thyroid function is associated with increased thyroglobulin level, which is known to be elevated in smoking pregnant women. This causes low immunity and increases vulnerability to infection (68). Maternal smoking may affect the body’s hormone levels by disturbing the gonadal axis, leading to congenital malformations (69, 70). Thus, further research is required to elucidate the mechanisms at work.

The incidence of cryptorchidism is related not only to genetic factors but also to parental environmental factors. Several studies (25, 29, 31, 43) have reported that maternal smoking during pregnancy increases the risk of cryptorchidism. Smoking causes impaired placental function, and cryptorchidism is associated with impaired placental functioning (31). Due to androgen-dependent male sexual differentiation, exposure to an environment that affects androgen homeostasis during fetal life may contribute to cryptorchidism (36). Changing levels of endogenous estrogen in smoking mothers may be associated with cryptorchidism in offspring. The report indicates that the levels of human chorionic gonadotropin and epidermal growth factor were lower in smokers than in non-smokers (71). A significant increase in cryptorchidism risk was also observed among sons whose mothers consumed oral contraceptives during pregnancy (54). There are pieces of evidence that the use of analgesics, such as paracetamol or paracetamol during pregnancy, especially in the first trimester, may increase the risk of cryptorchidism (72, 73). However, the potential mechanism of how drugs mediate cryptorchidism needs further study.

The relationship between maternal smoking with teratogenic hypospadias is uncertain. Tissue fusion is critical to the formation and function of organs and tissues during embryonic development, including the heart, neural tube, face, and urethra (74). Disruption of Shh and Fgf signals during urethral development leads to failure of urethral plate fusion (75). There are higher rates of congenital heart disease and cleft lip in infants of smoking mothers (24, 64). It has been reported that there may be associations between maternal heavy smoking and neural tube defects (59, 76). Studies have found that maternal smoking is related to birth defects of hypospadias (34). One study demonstrated that nicotine intake by smoking pregnant women modulates fibrosis by changing the function of fibrocytes (77). The fibrocyte growth factors play an indispensable role in the development of the urethral plate and the formation of hypospadias (78). A case reported in one study showed that hypoxia might result in hypospadias (79). However, the biological mechanisms underlying hypospadias caused by nicotine or other chemicals in tobacco remain unclear.

We observed the association between maternal smoking and renal defects, although a low number of studies was included. It has been reported that smoking during pregnancy is associated with renal malformation (49, 60). The reduction of ureteric branching and nephron number, through ureteric β-catenin signaling, is observed in the hypoxic kidney (80, 81). The prevalence rate of kidney malformations is so low that there are few reports about renal teratogenesis associated with maternal smoking. Due to the association between maternal smoking during pregnancy and spontaneous abortion or stillbirth (3, 5, 82), there is a weak association between maternal smoking during live birth and congenital kidney abnormalities. Possibly, the toxic effects of cigarette smoking during pregnancy clouded the teratogenic effect (7). Kidney malformations account for a small part of teratogenesis. Further studies are required with large sample size.

Paternal smoking leads to passive smoking; however, there were no reports on the harmful effects of passive smoking on urogenital teratogenesis. Our results suggested that passive smoking plays an important role in fetal development compared with active smoking. We speculate that non-smoking mothers may be more sensitive to alien smoking. Paternal smoking during pregnancy is associated with urethral stricture, possibly due to passive smoking (83). More research is needed to determine how passive smoking affects urogenital malformations.

The study had some limitations. First, the relationship between maternal medication use during pregnancy and urogenital malformations was not assessed. Some drugs may have potential etiological effects on urogenital development; however, this relationship cannot be assessed due to the limited number of cases. There are potential deviations from our results. Second, the association between the use of assisted reproductive technology in smoking mothers and urogenital malformations was not evaluated in our study. Demand for assisted reproductive technology increases as human male reproductive health declines. Some studies have reported that assisted reproductive technology increases the risk of congenital urogenital malformations. After a careful review of mothers’ characteristics, we found that information about assisted reproductive technology was not collected in the included studies. Thus, this relationship assessment was not performed. Confounding factors, such as alcoholic beverage use, age, education level, vegetarian diet, and history of infertility, had not always been considered in the study. Third, we did not find a dose-response effect between the quantity of smoking and the risk of urogenital teratogenesis. This might be due to the lack of continuous dose level measurements. Perhaps, studies with a higher sample size were used to assess associations between maternal heavy smoking and urogenital teratogenesis.

## Conclusion

An increased risk of congenital urogenital malformation is associated with smoking among pregnant women. There is a gradual increase in evidence of the harmful, comprehensive fetal toxic effects of smoking, warning pregnant women not to smoke at all during pregnancy.

## Data availability statement

The raw data supporting the conclusions of this article will be made available by the authors, without undue reservation.

## Author contributions

QZ designed the study, analyzed the data, and completed the manuscript. Z-CZ designed the study and assisted in drafting and revising the manuscript. X-YH and Z-ML independently searched and extracted the data. G-HW helped revise the manuscript. XL served as the corresponding author, provided financial support, and assisted with drafting and revising the manuscript. All authors read and approved the final manuscript.

## Abbreviations

CI: confidence interval
OR: odds ratio.
