# Supplementary material for: Maternal smoking during pregnancy and the risk of congenital urogenital malformations: A systematic review and meta-analysis
Source: Front Pediatr. 2022 Oct 3;10:973016. doi: 10.3389/fped.2022.973016 (PMC9575702; doi:10.3389/fped.2022.973016)
Supplement: Supplementary file 1 [file Table_1.DOCX]

| Study | Design | Region | Population | Matched or Adjusted Variables |
| --- | --- | --- | --- | --- |
| Himmelberger(1978) | Case-control | U.S. | 10523 live births from 12914 pregnancies | Age at pregnancy, smoking history, exposure to operating room, response to mailing |
| Evans (1979) | Cohort | U.K. | 67,609 pregnancies | No of cigarettes smoked daily, categories of all congenital malformations, singleton births |
| Christianson (1980) | Cohort | U.S. | 14,735 live births | Ethnic group, maternal  smoking status, primigravida, sex, gravidity, age, education |
| Shiono (1986) | Case-control | U.S. | 33,434 live births | Reproductive and medical history, use of tobacco, alcohol, contraceptives, study type |
| Malloy (1989) | Case-control | U.S. | 288,067 live births | Maternal race, age, marital status, parity, education |
| Mcbride (1991) | Case-control | U.K. | 732 live births | Mean birthweight, mean maternal age at first birth, No of mothers with medications during pregnancy, father's occupation during pregnancy |
| Mcdonald (1992) | Cohort | Canada | 89,317 pregnancies | Cigarettes per day, drinks per week, cups per day |
| De-Kun Li (1996) | Case-control | U.S. | 487 infants | Maternal age, marital status, race, education, age, family annual income, prenatal vitamin use, parity, prenatal alcoholic beverage use |
| Karin Kallen (1997) | Cohort | Swedish | 1,117,021 infants | Cigarettes per day, year of birth, maternal age, parity |
| Karin Kallen (2000) | Case-control | Swedish | 1,413,811 infants | Cigarettes per day, maternal education level, socio-economic status |
| Honein (2001) | Cohort | U.S. | 6,161,506 births | Maternal age, race, education |
| Pierik (2004) | Case-control | Netherlands | 8,698 births | Maternal age, parity height, education level, country of origin, good general health, folic acid supplements  in pregnancy, soy protein intake, lignan intake |
| Preiksa (2005) | Case-control | Lithuania | 1,204 births | Birth weight, preterm delivery, small gestational weight, gestational age |
| Morales (2006) | Cohort | Danish | 76,768 pregnancies | Age, parity, BMI, alcohol intake during study period, education, |
| Slickers (2008) | Case-control | U.S. | 943 pregnancies | Maternal age, race, parity, education, household income, gestational diabetes mellitus, maternal residence at delivery |
| Adams (2011) | Case-control | U.S. | 37,516 births | Year of birth, maternal race, age, education, number of previous live births, BMI, weight, parity |
| Leite (2014) | Cohort | Danish | 838,265 births | Year of birth, maternal age at delivery, maternal marital status, cigarettes per day |
| Dhalwani (2015) | Cohort | U.K. | 192,498 children | Age at conception, Townsend deprivation index score, BMI, maternal Characteristics |
| Groen In 't Woud S (2016) | Case-control | Netherlands | 2,701 pregnancies | Folic acid use, overweight, alcohol consumption, obesity, subfertility, diabetes mellitus， smoking |
| Davies (1986) | Case-control | U.K. | 318 boys | Maternal weight, height, social class, diabetes or glycosuria, any x-rays, ultrasound, cigarettes or drinking per day |
| Mori (1992) | Case-control | Japan | 204 mothers | Age, length of gestation, birth weight, duration of breast-feeding, parity |
| Berkowitz (1996) | Case-control | U.S. | 6,699 infants | Maternal age, race, marital status, education, BMI, clinic service, employment, history of infertility, parity, previous spontaneous abortions, use of clomid, perganol, analgesics |
| Moller (1996) | Case-control | Danish | 1,234 men | Years in school, professional education of mother or father, age, sibship sin |
| Akre (1999) | Case-control | Sweden | 20,017 children | Maternal age, parity, smoking, height, duration of gestation, history of subfertility, birth weight, preeclampsia |
| Biggs (2002) | Case-control | U.S. | 11,957 infants | Maternal marital status, age, race, trimester prenatal care began, prenatal smoking |
| Kurahashi (2005) | Case-control | Japan | 212 children | Maternal age, education, BMI, paternal age, education, vomiting of pregnancy, vaginal bleeding, gestosis |
| Thorup (2006) | Cohort | Denmark | 318 boys | Maternal smoking habits, age at surgery, birth weight |
| Virtanen (2006) | Case-control | Finnish | 1,288 boys | Maternal glucose metabolism class, premature delivery, age, preeclampsia, BMI |
| McGlynn (2006) | Case-control | U.S. | 12,534 boys | Maternal age, weight, height, paternal age, marital status, infertility history, hypertension, proteinuria, prior pregnancies |
| Jensen (2007) | Case-control | Danish | 5,716 infants | Maternal smoking related to pregnancy, cigarettes per day, nicotine content of cigarettes, age at delivery, paternal smoking status, age |
| Carbone (2007) | Case-control | Italy | 293 infants | Maternal age, education, parity, use of anti-abortion drugs, contraceptives used in the past, paternal age,weight |
| Main (2007) | Case-control | European | 280 boys | Maternal age, BMI, smoking, gestational age, diabetes, prematurity, infant blood samples |
| Mongraw-chaffin (2008) | Case-control | U.S. | 7,574 infants | Cigarettes per day, alcohol consumption,parity, race, age, caffeine consumption, BMI |
| Damgaard (2008) | Case-control | Danish-Finnish | 2,496 boys | Weight for gestational age, birth weight, parity, delivery, maternal age, BMI, smoking, social class, paternal age |
| Wagner-Mahler (2011) | Case-control | France | 6,246 boys | Testis position, at birth, duration of pregnancy, delivery, parents age, BMI, parity, family history, sterility treatment |
| Brouwers (2012) | Case-control | Netherlands | 829 boys | Educational level of parents, ethnic background of parents, prescriptive drug use, factors associated with subfertility, gestational and obstetric characteristics, familial history, maternal intake of folic acid |
| Bengt Kallen (1982) | Case-control | Sweden | 2,357 infants | Maternal age, parity, pregnancy length, birth weight, specific diagnoses, X-rayed during pregnancy |
| Kurahashi (2005) | Case-control | Japan | 95 mothers | Maternal age at delivery, maternal smoking, education, |
| Carmichael (2005) | Case-control | U.S. | 1,720 mothers | Maternal race-ethnicity, age, subfertility, education, state of residence, infant birthweight, maternal smoking |
| Brouwers (2007) | Case-control | Netherlands | 1,066 boys | Age, educational level of parents, native country of parents, increased maternal age at time of delivery, subfertility, parent’s life style, drug use, and occupational exposures |
| Giordano (2008) | Case-control | Italy | 292 births | Birthweight, mother’s age, education, BMI, smoking, food consumed, gynaecological diseases, father’s use of pesticides before pregnancy |
| Akre (2008) | Case-control | Denmark-Sweden | 719 mothers | Maternal age, BMI, height, education, history of irregular periods, contraceptive use at conception, daily cigarettes, passive maternal exposure to tobacco smoke during index pregnancy |
| Ormond (2009) | Case-control | U.K. | 961 infants | Maternal age, race, income, gestational age, birth weight, environmental tobacco smoke at home, maternal smoking |
| Brouwers (2010) | Case-control | Netherlands | 934 boys | Educational level of parents, ethnic background of parents, familial history, gestational and obstetric characteristics, prenatal DES exposure, factors associated with subfertility, prescriptive drug use, smoking |
| Estors Sastre (2019) | Case-control | Spain | 420 infants | Advanced maternal age, anti-abortives, consumption of other drugs, mother’s age, education, professional category, tobacco and alcohol consumption |
| Lili Yang (2022) | Cohort | China | 12,144,972 women | Maternal age at delivery, race, education, marital status, eclampsia, maternal smoking |
| Woods (2001) | Cohort | U.S. | 18,016 births | Maternal smoking, age, race, birth weight, diabetes, gestational age, |
| Parikh (2002) | Case-control | U.S. | 1,129 infants | Maternal age, race, education, alcohol and cigarette use, diabetes, hypertension, |
| Honein (2003) | Case-control | U.S. | 2,932 infants | Race, birth weight, gestational age, BMI, maternal smoking, maternal age, subfertility |
| Bengt Kallen (1988) | Case-control | Sweden | 564 infants | Previous spontaneous abortions, induced abortions, family situation, smoking |
| Karin Kallen (2002) | Case-control | Swedish | 1,413,811 infants | Maternal age, smoking, parity, education |
| Kjersgaard (2022) | Cohort | Danish | 85,923 infants | Maternal smoking, BMI, binge drinking episodes, caffeine, age at delivery, parity, education, year of birth |


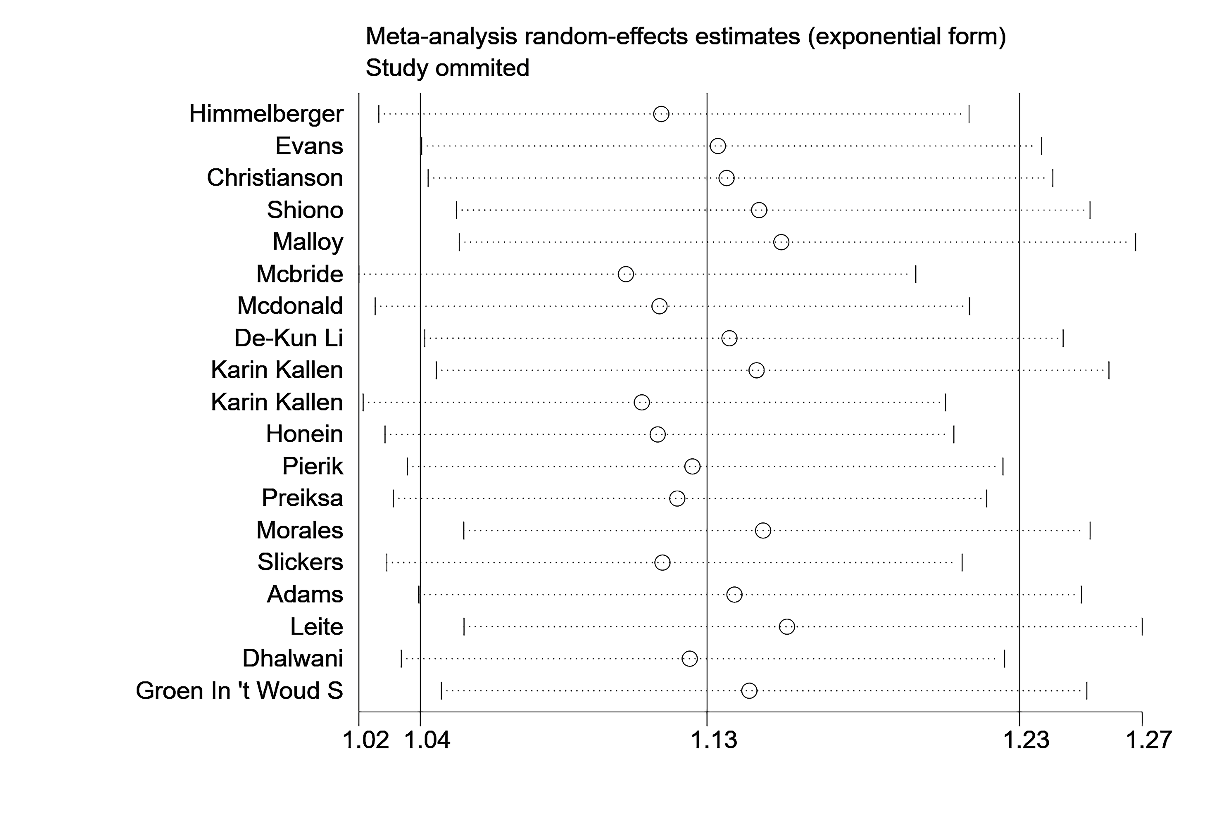
Table·1 The characteristics of included studies.

**Figure·8** Study sensitivity analysis based on the relationship between maternal smoking during pregnancy and the risk of congenital urogenital malformations.


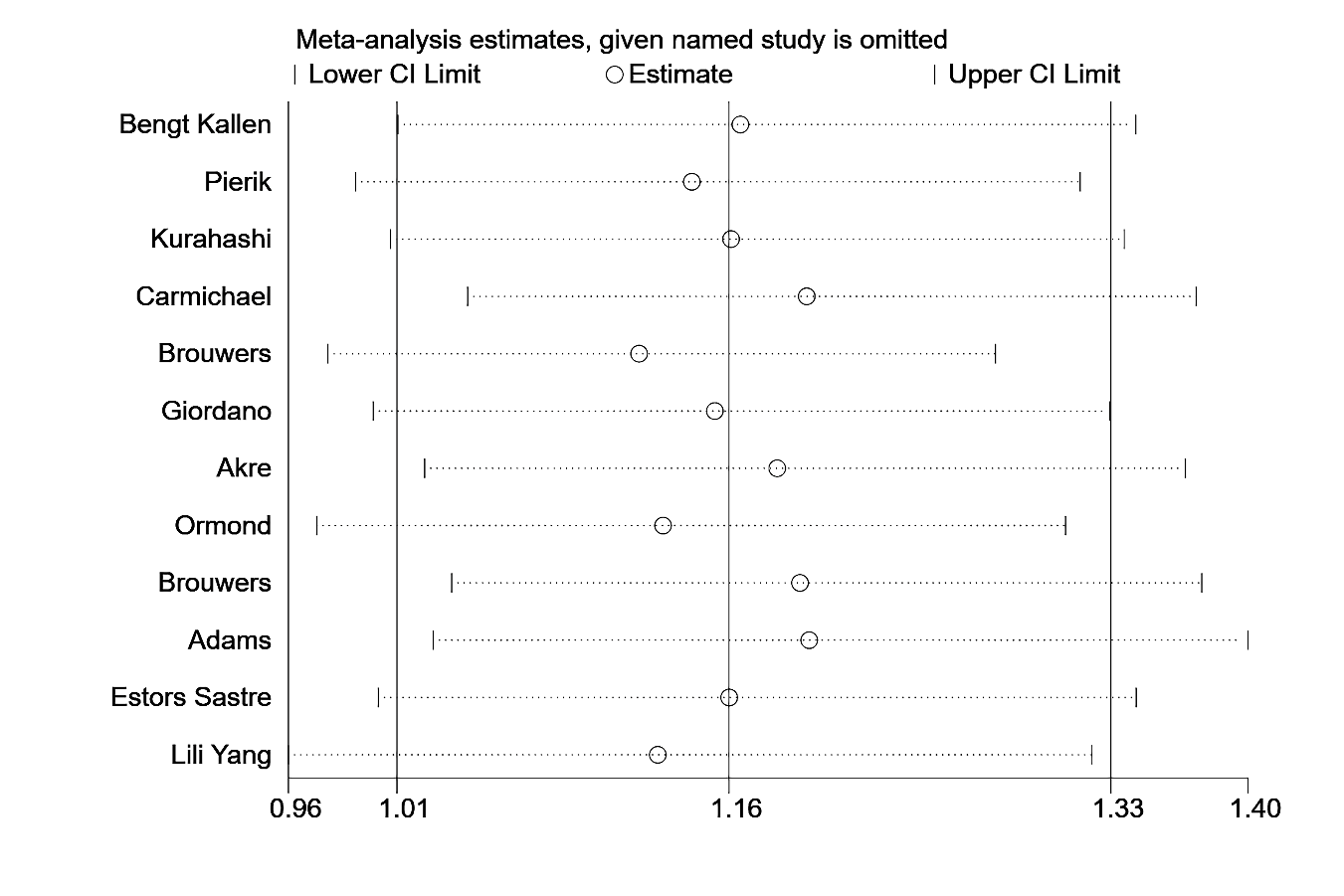

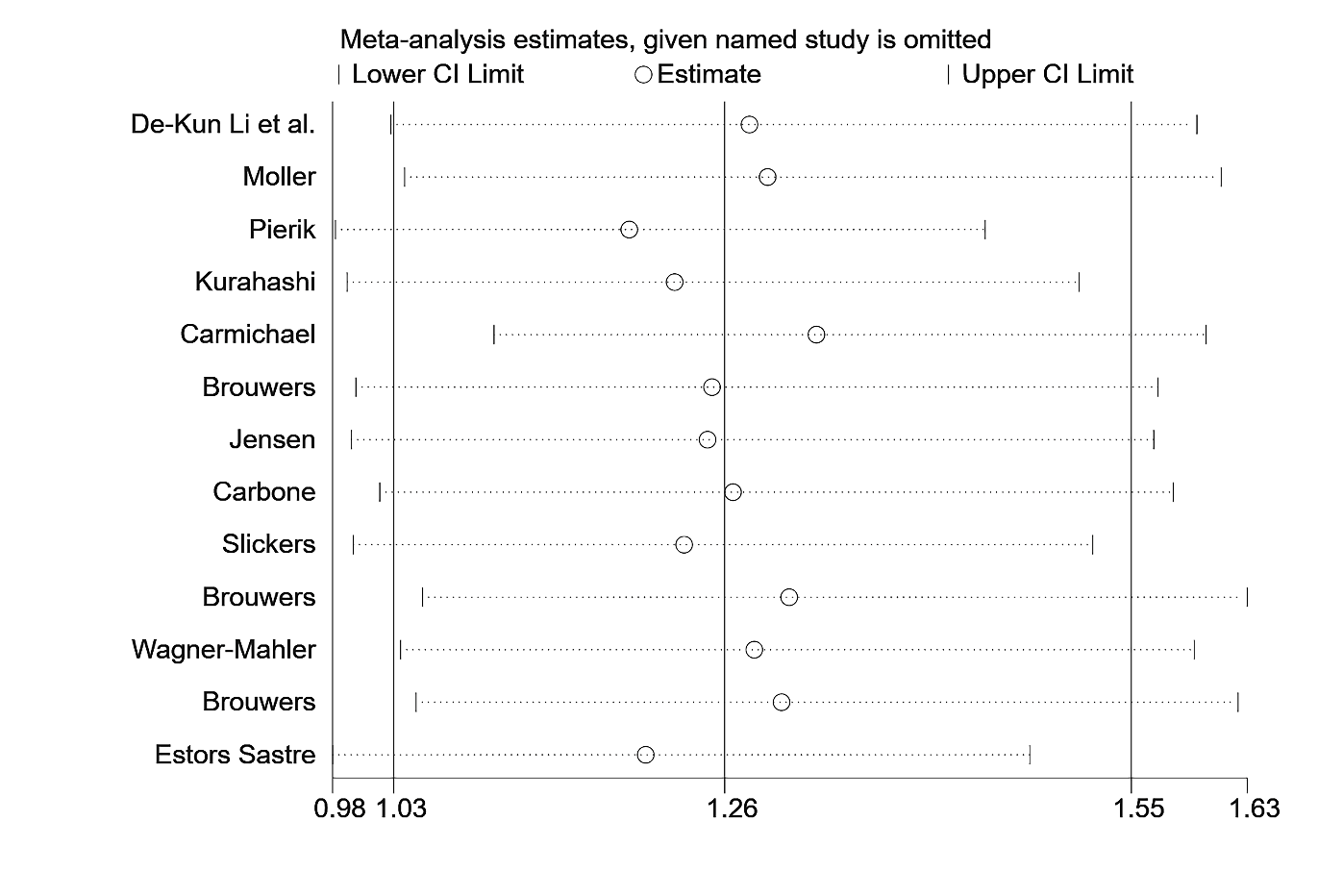
**Figure·9** A sensitivity analysis of included studies on the relationship between maternal smoking during pregnancy and the risk of hypospadias.

**Figure·10** Study sensitivity analysis based on the increased risk of congenital urogenital malformations when paternal smoking during a mother's pregnancy.

**
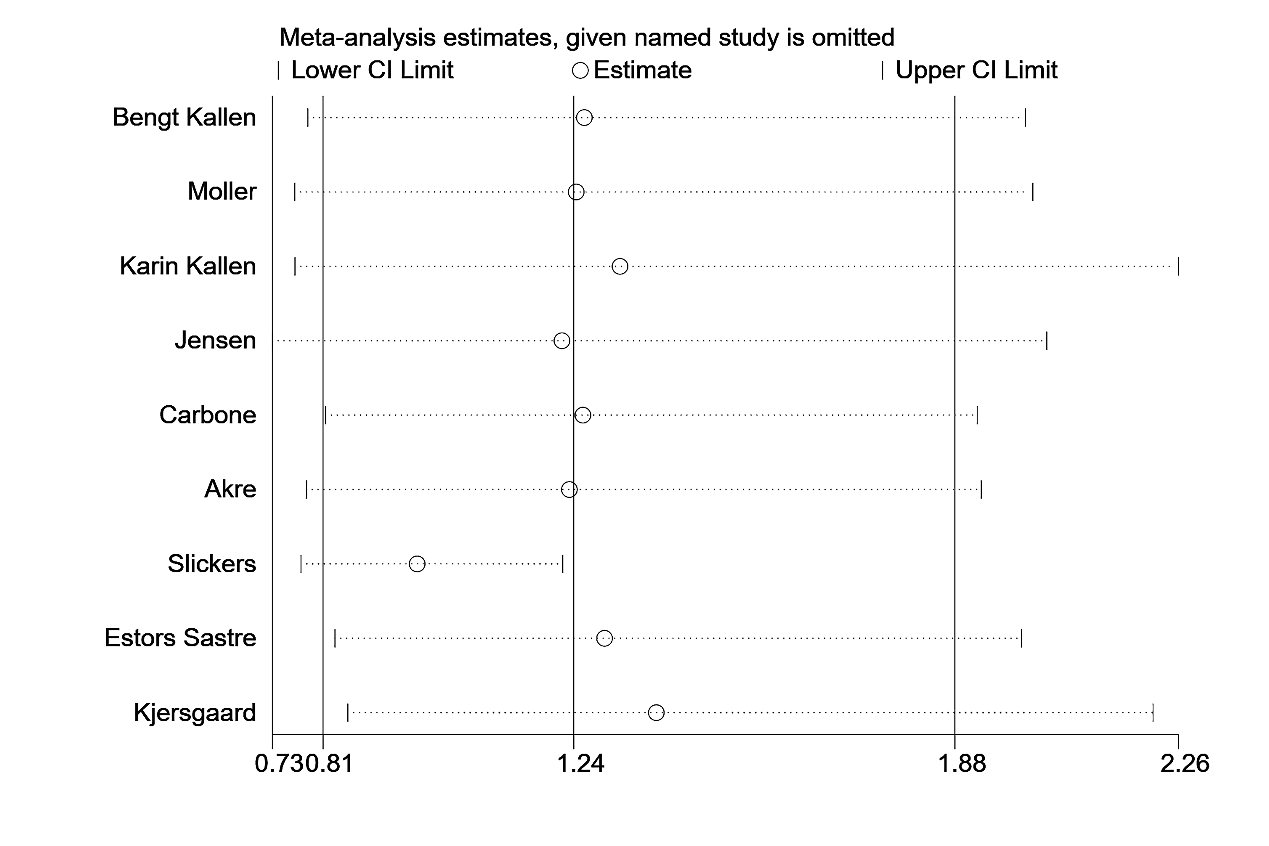
Figure·11** A sensitivity analysis of included studies on the nonsignificant association of >10 cigarettes/day when maternal smoking during pregnancy.
